# Disappearance of TBEV Circulation among Rodents in a Natural Focus in Alsace, Eastern France

**DOI:** 10.3390/pathogens9110930

**Published:** 2020-11-10

**Authors:** Laure Bournez, Gerald Umhang, Marie Moinet, Jean-Marc Boucher, Jean-Michel Demerson, Christophe Caillot, Léo Legras, Elodie Devillers, Yves Hansmann, Aurélie Velay, Céline Richomme, Sara Moutailler, Franck Boué

**Affiliations:** 1 Nancy Laboratory for Rabies and Wildlife, The French Agency for Food, Environmental and Occupational Health & Safety (ANSES), CS 40009, 54220 Malzéville, France; gerald.umhang@anses.fr (G.U.); Marie.Moinet@AgResearch.co.nz (M.M.); jean-marc.boucher@anses.fr (J.-M.B.); jean-michel.demerson@anses.fr (J.-M.D.); christophe.caillot@anses.fr (C.C.); leo.legras@hotmail.fr (L.L.); celine.richomme@anses.fr (C.R.); franck.boue@anses.fr (F.B.); 2 Unité Mixte de Recherche Biologie Moléculaire et Immunologie Parasitaire (UMR BIPAR), ANSES, INRAE, Ecole Nationale Vétérinaire d’Alfort, Paris-Est Sud, 94700 Maisons-Alfort, France; elodie.devillers@anses.fr (E.D.); sara.moutailler@anses.fr (S.M.); 3 Département en Maladies Infectieuses, Centre Hospitalier Universitaire de Strasbourg, 67000 Strasbourg, France; Yves.Hansmann@chru-strasbourg.fr; 4 Unité de Recherche 7290, Virulence Bactérienne Précoce: Fonctions Cellulaires et Contrôles de L’infection Aiguë et Subaiguë, Université de Strasbourg, F-67000 Strasbourg, France; 5 Laboratoire de Virologie, Centre Hospitalier Universitaire de Strasbourg, F-67091 Strasbourg, France; aurelie.velay@chru-strasbourg.fr; 6 Unité Mixte de Recherché Immunorhumathologie Moléculaire (UMR IRM_S) 1109, Université de Strasbourg, INSERM, 67000 Strasbourg, France

**Keywords:** tick-borne encephalitis virus, *Ixodes ricinus*, tick, small mammal, seroprevalence, endemic fadeout

## Abstract

Tick-borne encephalitis virus (TBEV) depends mainly on a fragile mode of transmission, the co-feeding between infected nymphs and larvae on rodents, and thus persists under a limited set of biotic and abiotic conditions. If these conditions change, natural TBEV foci might be unstable over time. We conducted a longitudinal study over seven years in a mountain forest in Alsace, Eastern France, located at the western border of known TBEV distribution. The objectives were (i) to monitor the persistence of TBEV circulation between small mammals and ticks and (ii) to discuss the presence of TBEV circulation in relation to the synchronous activity of larvae and nymphs, to the densities of questing nymphs and small mammals, and to potential changes in meteorological conditions and deer densities. Small mammals were trapped five times per year from 2012 to 2018 to collect blood samples and record the presence of feeding ticks, and were then released. Questing nymphs were collected twice a year. Overall, 1344 different small mammals (*Myodes glareolus* and *Apodemus flavicollis*) were captured and 2031 serum samples were tested for the presence of antibodies against TBEV using an in-house ELISA. Seropositive rodents (2.1%) were only found from 2012 to 2015, suggesting that the virus disappeared afterwards. In parallel, we observed unusual variations in inter-annual nymph abundance and intra-annual larval activity that could be related to exceptional meteorological conditions. Changes in the densities of questing nymphs and deer associated with the natural stochastic variations in the frequency of contacts between rodents and infected ticks may have contributed to the endemic fadeout of TBEV on the study site. Further studies are needed to assess whether such events occur relatively frequently in the area, which could explain the low human incidence of TBE in Alsace and even in other areas of France.

## 1. Introduction

Tick-borne encephalitis virus (TBEV) is a flavivirus causing a severe neurological disease in humans, with 2000 to 4000 cases reported yearly in the European Union [1]. In Western Europe, TBEV is maintained in nature through a cycle involving ticks—mainly *Ixodes ricinus*—and small mammals, especially those belonging to the genus *Apodemus* and *Myodes* [2]. Ticks can become infected while feeding on an infected host during the viraemic phase. Since the viraemia is considered to be short in a rodent host (2–9 days) and the transovarial transmission is very low, it is generally considered that the main mode of TBEV transmission to ticks is by co-feeding between infected nymphs and non-infected larvae when they feed close to each other on a small mammal host [3,4].

TBEV is less widely distributed than its tick vector and is only present in certain areas within the endemic region [5,6]. The distribution of TBEV is determined by specific biotic and abiotic conditions that favour the simultaneous feeding of larvae and nymphs on the same small mammals and therefore TBEV transmission to ticks by co-feeding [3,4,7,8]. Climate factors are the main determinants of the macro-scale distribution of TBEV in Western and Central Europe [8]. It has been hypothesised that a hot summer followed by a rapid drop in ground temperatures in autumn favours the synchronous emergence of larvae with nymphs in spring of the following year by inducing behavioural diapause in overwintering unfed immature ticks [7,8,9]. The absence of TBEV in the most western part of Europe such as in Western France could be related to the dyssynchronous activity of larvae and nymphs, with the peak of larval activity occurring in autumn whereas nymphs are mainly active in spring–early summer [7]. The micro-scale distribution of TBEV is more determined by the complex interaction of meteorological and biotic factors favouring habitat suitability for ticks and small mammals and the tendency of nymphs to feed on small mammals [10,11,12,13]. For instance, the relative abundance of competent hosts (small mammals) to incompetent but tick-amplifying hosts (deer—the main host for nymph and adult feeding) is an important driver of the number of nymphs feeding on rodents, and hence a key element in the persistence of TBEV [12,13]. TBEV thus persists under a limited set of biotic and abiotic conditions. Some natural TBEV foci might be unstable over time when the conditions for TBEV persistence are not fulfilled anymore, but such instability has rarely been observed in the field [14].

France is located on the western border of the known distribution of TBEV. Human clinical cases are mainly reported in the Alsace region of Eastern France, but the incidence is very low (0.5 autochtonous cases/100,000 inhabitants in Alsace). This incidence has slightly increased since 2016 however (2–8 autochtonous cases/year before 2016 vs. 6–26 autochtonous cases/year since 2016), and human cases have recently been reported in more areas than 15 years ago ([15], Velay A. and Y. Hansmann, unpublished data). Most human cases have been reported in forested areas of the Vosges, a fairly low mountain range located in the Western Alsace region. The Vosges mountains can provide a suitable habitat for the virus. Yet at the same time, given its location in Western Europe, its circulation in the Vosges range could be reduced by a higher variability in meteorological conditions, with a consequent decrease in transmission during co-feeding.

We had previously investigated the characteristics of the epidemiological cycle of TBEV in a Vosgian forest in Alsace (Murbach) from 2012 to 2014. The site was already known as a high-risk site for human contamination, and our investigations revealed that both the density of infected questing nymphs and the seroprevalence among small rodents were constant over time [16]. In the current study, our objective was to monitor the persistence of TBEV circulation between small mammals and ticks on the same site and to discuss it in relation to the synchronous activity of larvae and nymphs, to the densities of questing nymphs and small mammals, and to potential changes in meteorological conditions and deer density. From 2012 to 2014, the prevalence of TBEV in questing nymphs was very low from 0.03% to 0.24% requiring a very high number of ticks to analyse between 1400 and 7500 ticks to detect TBEV in 95% of cases [17]. Considering the high number of ticks that should be analysed to have a high probability of detecting TBEV and the the short viraemia in small mammals, testing small mammals for the presence of TBEV antibodies is the better approach for detecting the presence of TBEV with a lower cost [18,19]. We therefore evaluated TBEV presence by monitoring TBEV-seroprevalence among small mammals over three seasons. The three seasons were defined as: season 1 for early spring (beginning of nymph activity and small mammal reproduction), season 2 for the end of spring/early summer (peak of nymph activity and small mammal reproduction) and season 3 for end of summer/early autumn (decrease in nymph and small mammal abundance levels).

## 2. Results

### 2.1. Small Mammal Abundance

Over the 105 nights of capture, 3759 captures and recaptures of small mammals were recorded in total, corresponding to 1344 different individuals: 726 *Myodes glareolus*, 575 *Apodemus flavicollis*, 2 *Microtus agrestis* and 2 *Sorex* sp.

**Figure 1:**
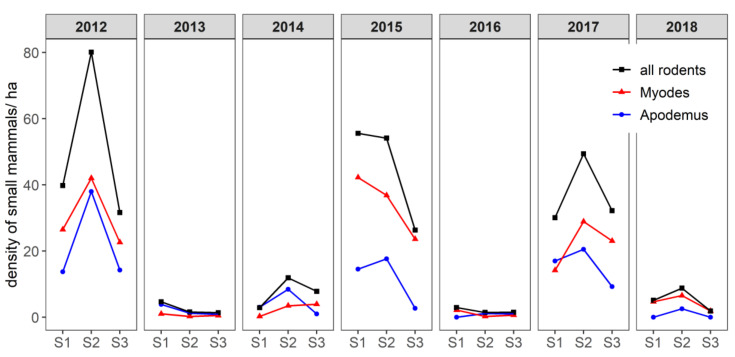
Estimation of the overall density of small mammals for two species in particular—the bank vole (*Myodes glareolus*) and yellow-necked mouse (*Apodemus flavicollis*)—per season and per year. Density is represented by the number of small mammals per hectare. S1: season 1 (early April–early May); S2: season 2 (early June–early July); S3: season 3 (early September–early October). For the purpose of visual clarity, confidence intervals are not shown.

The populations of bank voles (*M. glareolus*) and yellow-necked mice (*A. flavicollis*) had a synchronous inter-annual dynamic pattern (Figure 1). They peaked in 2012, 2015 and 2017 (>30 individuals/ha), crashed in 2013 and 2016 (<5 individuals/ha) and were intermediate in 2014 and 2018 (8–12 individuals/ha, Table 1). During the crash and intermediate phases, *A. flavicollis* was captured slightly more than *M. glareolus* except in 2018. During the peak phases, *M. glareolus* was slightly more abundant in 2012 and 2017 but twice as abundant in 2015 as *A. flavicollis*. Every year, the abundance of small mammals peaked in June and July (season 2). Abundance then decreased for both species, though bank voles were more abundant than yellow-necked mice in September–October (season 3).

### 2.2. Prevalence of Tick Infestation in Small Mammals

The proportion of rodents infested at least once by ticks significantly varied between years (Chi^2^ = 206, df = 6, *p*-value < 0.001), ranging from 20.3% [95% CI: 16.0–25.3] to 46.2% [95% CI: 41.1–51.3] for years of peak rodent abundance and from 69.2% [95% CI: 38.6–90.9] to 95% [95% CI: 73.9–99.9] for years of low and intermediate abundance (Table 1). It varied according to season and year (Table 1). Concerning the years of peak abundance, the prevalence of tick infestation was significantly higher in season 2 than season 3 in 2012 (44.0% vs. 23.1%, Chi^2^ = 16, *p*-value < 0.001) and 2015 (35.4% vs. 18.1%, Chi^2^ = 12, *p*-value < 0.001) but in 2017 it was the opposite (4.7% vs. 31.9%, Chi^2^ = 55, *p*-value < 0.001, Table 1). Few small mammals were infested by ticks during June and July in 2017 (4.9% [95% CI: 2.5–8.4]) compared to 2012 and 2015 (respectively 45.1% [95% CI: 39.8–50.5] and 36.0% [95% CI: 29.9–42.5], Table 1). The prevalence of tick infestation was significantly higher for yellow-necked mice than for bank voles only in season 2 of 2012 and 2015 (2012: 58.8% vs. 29.7%, Chi^2^ = 30, *p*-value < 0.001; 2015: 54.2% vs. 25.8%, Chi^2^ = 18, *p*-value < 0.001). In other seasons and years, there was no significant difference in the prevalence of tick infestation between species (Table 1).

### 2.3. Detection of TBEV Antibodies in Small Mammals

We analysed 2031 serum samples: 1249 samples from 726 *M. glareolus* and 782 from 575 *A. flavicollis*. Overall, 2.1% of individuals were TBEV-seropositive. This proportion decreased gradually over time with 4.7% [95% CI: 2.9–7.2] in 2012 (n = 422), 2.7% [95% CI: 0.1–14.2] in 2013 (n = 36), 1% [0.1–5.2%] in 2014 (n = 104), 1.9% [95% CI: 0.1–4.0] in 2015 (n = 359), 0% [95% CI: 0–16.8] in 2016 (n = 30) and 0% [95% CI: 0–1.0] in 2017 (n = 362, Table 1). The minimum TBEV seroprevalence that could be detected in rodents in 2017 with a probability of 95%, given the overall sampling size, was 0.8%. This suggests that the overall TBEV seroprevalence in rodents was less than 0.8%.

No statistical difference was found in seroprevalence between seasons in 2012 (3.2–5.2%, Fisher’s exact test, *p*-value =0.53) and 2015 (1.3–1.6%, Fisher’s exact test, *p*-value =1, Table 1), with TBEV antibodies found every month during the April to October sampling periods. There was no significant difference between species in 2012 (bank vole 3.4% vs. yellow-necked mice 4.0%, Chi^2^ = 0.02, *p*-value = 0.9) and 2015 (bank vole 1.6% vs. yellow-necked mice 0.8%, Fisher’s exact test, *p*-value = 1).

### 2.4. Questing Tick Densities

**Figure 2:**
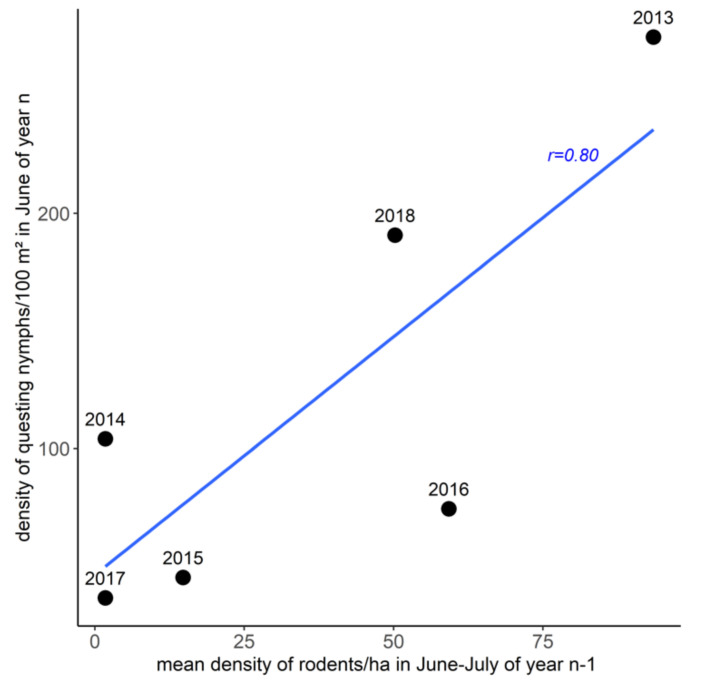
Correlation between the density per 100 m^2^ of *Ixodes* sp. Questing nymphs in June (year n) according to the maximal density of small mammals in June–July of the previous year (year n-1). The text above each point indicates the corresponding year (n).

Overall, 4413 *Ixodes* sp. Nymphs were collected. Questing *Ixodes* sp. Nymph densities varied between years and were much higher in early June in 2013 and 2018 (respectively 190 and 280 nymphs/100 m^2^) than in other sampling years (from 30 to 100 nymphs/100 m^2^; Table 2). The peak density of questing nymphs in a given year was positively correlated with the mean density of small mammals in June–July of the previous year (Figure 2; Pearson’s *r* = 0.80, *p*-value = 0.05). This correlation was higher without the value of 2016 (Pearson’s *r* = 0.94, *p*-value = 0.02).

## 3. Discussion

### 3.1. Synchronous Activity of Larvae and Nymphs in 2012–2018

By using the prevalence of tick infestation of small mammals as an indicator of larval activity, we observed synchronous activity of nymphs and larvae on the study site in spring to early summer in all years but 2017. The seasonal abundance of small mammals was also the highest at the end of spring to early summer. This confirmed the results previously described on the same study site over a shorter timespan [16]. The synchronous activity of larvae and nymphs, as observed at other sites at risk for TBEV [11,20,21], is usually recognized to be essential for the maintenance of TBEV by favouring transmission through co-feeding [3,4,7,22]. Some theories suggest that this synchrony would occur if associated with a rapid drop in temperatures in autumn leading unfed larvae to enter behavioural diapause during the winter and to be mainly active in spring, at the same time as nymphs [7,9]. However, in 2017, we observed that larval infestation of rodents was very low in June–early July, and the proportion of rodents infested by larvae in the autumn was no different from the other years. The cooling rate in autumn 2016 was no different from the other years (Figure S1a). Nevertheless, 2017 was marked by unusually warm temperatures from the end of February to mid-April, followed by unusually cold temperatures at the end of April with snowfall (Figure S1b). In response to rising temperatures during February and March, most engorged females might have started hatching their eggs and most unfed larvae might have become active by March. The drop in temperatures in April (from 16 °C to −5 °C) could therefore have induced high mortality in hatching eggs or unfed larvae. Indeed, it has been shown experimentally that the frequency of temperature variations between below and above activity thresholds impairs the survival of questing ticks [23,24], with a lower survival in larvae than nymphs [23]. More studies are necessary to confirm this hypothesis. However, this finding clearly shows that the usual activity pattern of ticks observed during this study can exceptionally change with unusual meteorological conditions.

### 3.2. Disappearance of TBEV on the Studied Site

TBEV antibodies were detected in rodents each year from 2012 to 2015 but not after 2016. During the years when small mammals were abundant, antibodies were detected in each season in 2012 (3.2–5.2% of individuals per season) and 2015 (1.3–1.6% of individuals per season), but not in 2017. The TBEV-seroprevalence in small mammals decreased over time from 2012 to 2015, although the difference between 2012 and 2015 was not significant, and was null in 2016 and 2017. This may reflect either a local disappearance of TBEV after 2015 or a low, undetected, exposure of rodents to TBEV, with a seroprevalence below 0.8% in 2017. Our previous results showed that the density of infected nymphs was constant from 2012 to 2014 [16]. The ticks collected since 2015 were also tested for TBEV, but the number of tested samples was too low to have a high probability of detecting TBEV if the virus was present (Supplementary Material 1). The number of infected nymphs questing for a host in the environment probably decreased over time and was at some point after 2014 too low to maintain the circulation of TBEV.

Numerous reasons can explain the virus fade-out observed in the present study. The basic reproduction number *R_0_* of TBEV and hence the persistence of TBEV strongly depends on the ratio of feeding ticks to rodents (i.e., intensity of the aggregation of ticks on rodents), the number of co-feeding groups of larvae and nymphs, and the proportion of ticks feeding on competent hosts, which in turn depends on the density of other hosts, especially deer [3,13,22]. The dissociation of larvae and nymph activity observed exceptionally in 2017 cannot explain the virus’s disappearance here since TBEV antibodies were not detected in small mammals in 2016 and 2017, before and at the same time as this event. We cannot exclude that we missed seropositive rodents in 2016, given their low abundance, but the absence of seropositive animals sampled in 2017 leads us to think that TBEV disappeared from the study area before 2017. Indeed, nymphs were still active in spring 2017 and, if present, TBEV-infected nymphs could still have induced an antibody response in the small mammals on which they were feeding in 2017, even in the absence of larvae. However, the frequency of such unusual meteorological events may influence the long-term maintain of TBEV in this area.

The disappearance of TBEV on the site might be due to an overall decrease over time of the number of ticks—mainly nymphs—feeding on rodents. Instead, a higher number of nymphs may have fed on alternative but not TBEV-competent hosts such as cervids, which are among the preferred hosts for nymphs [25]. The study site hosts a high density of red deer (*Cervus elaphus*) and roe deer (*Capreolus capreolus*), with around 10 animals/100 wooded ha (source: DDT 68, official services in charge of hunting for the Haut-Rhin *département*). They became even more abundant throughout the study period (Figure S2). The increase in deer populations may have increased the rate of encounters between ticks and deer, and hence decreased those between ticks (especially nymphs) and rodents and the number of ticks feeding on rodents.

The disappearance of TBEV might also be partly related to an increase in the mortality of larvae and nymphs in the environment due to unusual meteorological conditions. The densities of questing nymphs were at their lowest levels for four consecutive years from 2014 to 2017, reaching its lowest level in 2017. This was in contrast with the density of small mammals. Although the density of questing nymphs was generally observed to spike the year after an abundant year for small mammals, this was not the case in 2016. Unusual meteorological conditions, such as the warm and dry summer in 2015 with a saturation deficit higher than 10 mmHg over several days (Figure S1b–e), might have decreased the survival of questing larvae, fed larvae or questing nymphs [26,27]. This unexpected event may also have contributed to decreasing the overall density of nymphs questing in the environment and then to decreasing the number of infected nymphs feeding on rodents. Moreover, the unusually hot temperatures observed in 2015 might also have induced a drop in ticks’ virus titre. Indeed, it has been shown experimentally that long-term exposure to high temperatures, e.g., 37 °C or 22 °C with a gradually decreasing humidity over 2 months, resulted in a drop in tick virus titres ([28,29] cited by [30]).

TBEV dynamics on this site might also be very sensitive to stochastic processes influencing the probability of contact between infected nymphs and rodents that can sometimes lead to virus extinction via endemic fadeouts [31], given the marked fluctuation in the rodent population and the low tick infestation of rodents. The number of ticks per rodent on this site observed in 2012 (a year with a high rodent density) and 2013 (a year with a low rodent density) was low (with a median of fewer than five ticks/rodents infested by ticks [16]) compared to other sites in Central Europe where TBEV is present (where the median ranges from ten to 80 ticks per infested rodent [11,21,32]). With the low number of feeding ticks per rodent observed in this study and the high density of deer present on the site, even a small variation in the number of questing nymphs or deer density could disrupt the virus transmission cycle by decreasing the number of infected nymphs feeding on rodents [13]. Moreover, under these conditions, the very low rodent densities observed some years may sometimes be below the threshold needed to maintain the TBEV cycle [13] despite a higher aggregation of ticks on rodents during these years. The composition of the rodent population could also influence the annual R_0_ and the persistence of TBEV. Experimentally, yellow-necked mice are more competent for transmitting TBEV than bank voles [33]. This species has been found to be generally more infested by ticks than bank voles [34,35,36], which is a similar finding to our results. However, the probability of infected nymphs feeding on yellow-necked mice probably depends on the overall abundance of small mammals and the proportion of this species among the small mammal community, as suggested for other tick-borne pathogens [37]. Therefore, the lower abundance of yellow-necked mice in some years compared to bank voles (e.g., in 2015) might contribute to the overall decrease in infected larvae produced. Moreover, the probability of contact between infected nymphs and yellow-necked mice could be highly variable in the years of low rodent density. In conclusion, changes in the density of questing nymphs and/or in the density of deer associated with the natural stochastic variations in the frequency of contact between rodents and infected ticks could have contributed to the endemic fadeout of the virus on our site. Mathematical modelling integrating stochastic effects would be useful to assess these effects on the persistence of TBEV.

The disappearance of the virus is probably very local. The records of clinical cases of tick-borne encephalitis virus in humans (Figure S3) suggest that the virus probably continues to circulate in the rest of the Guebwiller valley, where our study site was located, although it is difficult to pinpoint exactly where the victims were bitten by infected ticks. Two patients were reported to have been bitten in this valley in 2016, two in 2018 and two in 2019, including one in Murbach (the same municipality as the study site) in 2018. In the nearby Munster valley, three cases were reported in 2016 and two in 2019. The different micro-meteorological conditions and the density of rodents, deer and ticks could explain the continued circulation of TBEV in nearby areas. These observations suggest that the cold temperatures of April 2017, which probably led to reduced larval activity in spring 2017 on our site, was only extreme enough in areas over a certain altitude (>600 m like our site) to decrease the number of infected questing ticks.

## 4. Conclusions

This longitudinal study shows that TBEV probably disappeared from the study site in Alsace during the seven years of this research. In parallel, we observed unusual variations in inter-annual nymph abundance and intra-annual larval activity that could be related to exceptional meteorological conditions. The relative densities of the tick host community also changed significantly. These variations may have disrupted the fragile TBEV transmission cycle on this site by decreasing the number of ticks and infected nymphs feeding on rodents. Further investigations are needed to explore whether TBEV circulation in Alsace regularly faces stochastics fadeouts, which could partly explain the low incidence of TBEV in Alsace compared to other European regions further east.

## 5. Materials and Methods

### 5.1. Study Area

The study took place in the same area as described in [16]. Briefly, the study site covers an area of 4 ha at Murbach (altitude of 630 m) in Guebwiller valley in the French region of Alsace (47°55′03 N, 07°08′46 E). The site is covered by mixed forests classified as *Asperulo-Fagetum* beech forests. Most human TBE cases in Alsace were reported between 2006 and 2012 in this area.

There is a high density of both roe and red deer in the area. The exact density of cervids on this site is unknown but it was probably around ten animals per 100 wooded ha in 2018 (source: DDT 68, official services in charge of hunting in the Haut-Rhin département). The Kilometric Abundance Index (i.e., the mean number of deer seen per kilometre across all transects walked within the area in a given time interval [38]) and the hunting bag in the studied or neighbouring areas suggest that the density of cervids has increased 1.2–1.3 times from 2012 to 2018 (Figure S2).

The climate is mountainous, with cold winters (coldest months from December to February with a mean temperature of 1.2°C) and mild summers (mean of 18.3°C in July and August). The meterological data from the period of the study (2012 to 2018) are described in Supplementary Material 2. Briefly, the autumnal cooling rate estimated by linear regression of the average daily temperature from 1st August to 31st October was the lowest in 2014 given the mild temperatures in August 2014 (Figure S1a). 2015 was marked by a particularly hot and dry summer and autumn, reaching the highest maximal temperature recorded since 1986 in July and August (35.5 °C) (Figure S1b–d). Consequently, the saturation deficit, which integrates temperature and relative humidity to derive a measurement of the atmosphere’s drying power [10,39], exceeded 10 mmHg for 14 days in the summer of 2015 (Figure S1e). This is a threshold above which tick survival may decrease [26,27]. 2017 was marked by a hot and dry early spring, with daily temperatures reaching the threshold of 10 °C for larval questing activity [27,39] at the end of March. Temperatures dropped rapidly after mid-April with snowfall (minimum of −5 °C) (Figure S1b–d).

### 5.2. Small Mammals: TBEV-Seroprevalence and Prevalence of Tick Infestation

We monitored the proportion of small mammals harbouring TBEV antibodies per season and per year to assess the presence of TBEV circulation. Since the phenology of questing nymphs is generally stable over time, with peak activity always occurring in spring, the synchronous activity of larvae and nymphs was studied by monitoring only the period of larval activity. This monitoring involved studying the proportion of small mammals harbouring feeding ticks, since >95% of ticks on small mammals were larvae in 2012 and 2013 [16].

Small mammals were trapped five times per year, during April and the first week of June, July, September and October from 2012 to 2018. The trapping grid consisted of 196 live-traps (14 × 14 Ugglan special nr. 3, Grahnab, Sweden) with 15 m spacing, covering a total area of 4 ha. For each session, traps were set for three consecutive nights and baited with carrots and sunflower seeds. Trapped small mammals were individually marked with a transponder (Vétérissimo Mini RWI-I, Vethica, France). Blood samples were taken once per session through the retro-orbital sinus. Species and tick presence were recorded except in April 2017, when no tick presence data were recorded in order to decrease rodents’ handling time because of the snowfall and cold temperatures. Animals were then released at the point of capture. Blood samples were kept at +4 °C until being brought back to the laboratory. There, blood samples were centrifuged at 5000 rpm for 5 min and the serum samples obtained were stored at −20 °C until tested for the presence of TBEV antibodies.

These serum samples were screened for the presence of TBEV antibodies using an in-house indirect ELISA developed to capture specific IgG antibodies. Purified SNAP-TBEV EDIII recombinant protein (encoding the soluble ectodomain (residues E293 to E399) from TBEV strain Kumlinge A52) and non-relevant SNAP were used to perform an ELISA as previously described in [16]. Serum was considered positive when the duplicate mean OD values read for SNAP-TBEV EDIII minus the mean OD values read for SNAP was higher than 0.100 OD.

Given the small number of seropositive small mammals, we grouped data into three seasons as in [16]: (1) season 1 (early spring) for the captures of small mammals in April representing the beginning of the periods of nymph activity and small mammal reproduction; (2) season 2 (end of spring/early summer) for the captures of early June and July representing peak nymph activity and reproduction of small mammals; and (3) season 3 (end of summer/early autumn) for the captures of early September and October representing the end of the nymph activity and small mammal reproduction periods.

Rodent density per season was calculated using the mean of rodent density estimated for the corresponding months. Since the captures were carried out on three consecutive days per session, we considered that the population was closed for each session and we calculated the density of rodents per session by using the standardised Schnabel closed population method that takes into account multiple marking occasions [40].

For each season, we listed each individual small mammal captured along with its tick infestation status and its TBEV status (seropositive or negative). An individual was considered to be infested by ticks in season 2 (respectively season 3) if it was found to be infested by at least one tick in June and/or July (respectively in September and/or October). Similarly, an individual found to be infested by ticks in at least one of the five trapping sessions of the year (from April to October) was considered infested by ticks in that year. An individual was considered to be TBEV-seropositive in season 2 (or season 3, respectively) if it was found to be TBEV-seropositive in June and/or July (or in September and/or October, respectively). An individual found to be TBEV-seropositive in at least one of the five trapping sessions of the year (from April to October) was considered TBEV-seropositive in that year. Exact 95% CIs were calculated using the binomial distribution. If no TBEV antibodies were detected in rodents in a given year, we calculated the minimum seroprevalence of TBEV that could be detected in rodents with a probability of 95% given the sample size by applying the formula proposed by Cannon [17].

### 5.3. Questing Tick Sampling

Since nymph activity peaked in spring each year, we only measured the density of questing nymphs twice a year to compare the density of questing nymphs between years: during the first week of June during the peak of questing nymphs as observed in 2011–2014 [16], and during the first week of September during the secondary peak of questing nymphs. Questing nymphs were counted and collected by dragging a 1-m^2^ white blanket over the same area as the rodent trapping grid. The trapping grid was divided into 16 smaller grids, and the blanket was dragged over three different 10-metre-long transects within these 16 grids. In all, 48 different transects totalling a surface area of 480 m^2^ were investigated within the 4-ha study site. All the questing nymphs were washed in 70% ethanol, dried and stored at −80 °C. They were then identified at genus level.

The density of questing nymphs in June and September was expressed as the mean number of ticks per 100 m^2^ per month, and was compared between years. The density of questing nymphs in June was considered as a proxy for the maximum density of nymphs reached during the year.

Since inter-annual variation in the density of questing nymphs is mainly influenced by the variation in small mammal abundance with a one-year lag, and to a lesser extent by the variation in meteorological conditions [41,42], we tested the correlation between the density of questing nymphs during the peak phase in June and the mean density of small mammals in June-July of the previous year by calculating the Pearson correlation coefficient. The values that differed the most from the expected linear relationship were identified and considered to be mostly influenced by meteorological conditions.

Statistical computations were performed in R 3.5.0 [43].

### 5.4. Ethical Statement

The experimental protocol with small mammals complied with EU Directive 2010/63/EU and was submitted to and approved by the French Ministry of Research (APAFIS no. 2015120215112678). All efforts were made to minimise animal suffering. The species studied are neither protected nor included in the IUCN Red List of threatened species in France. The animal trapping took place with permission from the landowners.

## Figures and Tables

**Table 1:** Proportion of small mammals infested by ticks or TBEV-seropositive according to year and season (season 1: April; season 2: early June and July; season 3: early September and October).

Year	Season	No. Infested by Ticks/No. Inspected (%)			No. TBEV-Positive/No. Tested (%)		
		Tot	*Myodes*	*Apodemus*	Tot	*Myodes*	*Apodemus*
2012	Season 1	37/104 (35.6)	24/72 (33.3)	13/32 (40.6)	4/95 (4.2)	2/65 (3.1)	2/30 (6.7)
	Season 2	158/359 (44.0)	54/182 (29.7)	104/177 (58.8)	11/349 (3.2)	5/178 (2.8)	6/171 (3.5)
	Season 3	28/121 (23.1)	22/93 (23.7)	6/28 (21.4)	5/97 (5.2)	4/75 (5.3)	1/22 (4.5)
2013	Season 1	2/16 (12.5)	0/4 (0)	2/12 (16.7)	0/16 (0)	0/4 (0)	0/12 (0)
	Season 2	11/11 (100)	3/3 (100)	8/8 (100)	1/10 (1.0)	0/3 (0)	1/7 (14.3)
	Season 3	8/10 (80.0)	2/3 (66.7)	6/7 (85.7)	0/10 (0)	0/3 (0)	0/7 (0)
2014	Season 1	4/7 (57.1)	1/1 (100)	3/6 (50)	0/7 (0)	0/1 (0)	0/6 (0)
	Season 2	65/69 (94.2)	17/20 (85.0)	48/49 (98.0)	0/60 (0)	0/16 (0)	0/44 (0)
	Season 3	11/20 (55.0)	8/12 (66.7)	3/8 (37.5)	1/20 (5.0)	1/12 (8.3)	0/8 (0)
2015	Season 1	5/129 (3.9)	4/89 (4.5)	1/40 (2.5)	1/79 (1.3)	1/51 (2.0)	0/28 (0)
	Season 2	87/246 (35.4)	42/163 (25.8)	45/83 (54.2)	3/220 (1.4)	2/143 (1.3)	1/77 (1.3)
	Season 3	25/138 (18.1)	19/123 (15.5)	6/15 (33.3)	2/122 (1.6)	2/109 (1.8)	0/13 (0)
2016	Season 1	8/8 (100)	6/6 (100)	2/2 (100)	0/10 (0)	0/8 (0)	0/2 (0)
	Season 2	9/9 (100)	2/2 (100)	7/7 (100)	0/9 (0)	0/2 (0)	0/7 (0)
	Season 3	2/7 (28.6)	2/4 (50.0)	0/3 (0)	0/5 (0)	0/2 (0)	0/3 (0)
2017	Season 1	77 *	41 *	36 *	0/75 (0)	0/40 (0)	0/35 (0)
	Season 2	12/253 (4.7)	7/142 (5.0)	5/111 (4.5)	0/241 (0)	0/136 (0)	0/105 (0)
	Season 3	53/166 (31.9)	33/106 (31.3)	20/60 (33.3)	0/162 (0)	0/106 (0)	0/56 (0)
2018	Season 1	10/20 (50.0)	9/18 (50.0)	1/2 (50.0)	0/20 (0)	0/18 (0)	0/2 (0)
	Season 2	39/45 (86.7)	28/32 (87.5)	11/13 (84.6)	0/42 (0)	0/31 (0)	0/11 (0)
	Season 3	6/6 (100.0)	6/6 (100.0)	0	0/6 (0)	0/6 (0)	0/6 (0)

Shaded rows correspond to a season with at least one TBEV-seropositive animals. * Tick presence on the animals was not recorded, so only the number of animals captured is reported here. Data from 2012 to 2014 were published in [16]. No. = Number.

**Table 2:** Density of *Ixodes* sp. Questing nymphs and adults per 100 m^2^ in early June and early September in a 4-ha area in Murbach forest, Guebwiller valley, France. Data from 2012 to 2014 were published in [16].

Year	Density of Questing Ticks (/100 m^2^)	
	Early June	Early September
2012	86.5	6.3
2013	275.0	39.0
2014	104.2	6.0
2015	45.2	27.7
2016	74.4	5.6
2017	36.5	19.6
2018	190.8	2.7

## Supplementary Materials

The following are available online at https://www.mdpi.com/2076-0817/9/11/930/s1, Supplementary Material 1: TBEV detection in questing ticks including Table S1: TBEV detection in questing ticks collected from 2015 to 2018. Supplementary Material 2: Meteorological data from 2012 to 2018 including Figure S1a: Autumnal cooling rate of temperatures estimated by linear regression of the average daily temperatures from 1st August (Julian day 214) to 31st October (Julian day 305); Figure S1b: Average daily temperatures per Julian day from 2012 to 2018; Figure S1c: Boxplot of the average daily temperatures per month from 2012 to 2018; Figure S1d: Boxplot of the average relative humidity per month from 2012 to 2018. Figure S1e: Boxplot of the average saturation deficit per month from 2012 to 2018. Figure S2. Trends of the relative density indices of deer populations in two adjoining hunting units (called GIC), GIC14 including the Murbach study area and GIC6 including the Munster valley. Figure S3: Distribution of human tick-borne encephalitis cases in Alsace from 2017 to 2019.
