# Supplementary material for: Disappearance of TBEV Circulation among Rodents in a Natural Focus in Alsace, Eastern France
Source: Pathogens. 2020 Nov 10;9(11):930. doi: 10.3390/pathogens9110930 (PMC7697581; doi:10.3390/pathogens9110930)
Supplement: Supplementary file 1 [file pathogens-09-00930-s001.zip › pathogens-980068-supplementary.pdf]

## Supplementary Materials

### Supplementary Material 1. TBEV detection in questing ticks.

Questing ticks were analysed to detect TBEV RNA. Adult ticks were analysed individually and nymphal ticks were analysed in pools of one to five ticks. RNA were extracted as described in [5] and were screen for TBEV by real-time RT-PCR targeting a 3′ non-coding region of the TBEV genome with specific primers and probes [6] as described in [5].

As TBEV infection prevalence in ticks is usually lower than 1% in Alsace [5,7,8], prevalence in ticks was expressed as the minimum infection rate per 100 tested (MIR), based on the assumption that a single tick was positive within a positive pool. Exact 95% CIs were calculated on the basis of binomial distribution. When no TBEV was detected in a sample of ticks, we calculated the minimum prevalence of TBEV that could be detected in ticks with a probability of 95% given the sample size used by applying the formula proposed by Cannon [9].

Results of the ticks collected in 2012-2014 have already been presented in [5]. No ticks were found positive to TBEV from 2015 to 2018 (Table S2). The minimal prevalence that could be detected in questing nymphs with a probability of 95% given the sample size varied from 1.4% to 11% according to the sampling period. Given the TBEV prevalence of 0.03% to 0.24% in questing nymphs, the number of ticks tested was not enough to detect TBEV.

**Table S1.** TBEV detection in questing ticks collected from 2015 to 2018.

| Year | Month | No of nymphs | No of females | No of males | No of TBEV-positive ticks | Minimal prevalence detectable in nymphs given the sample size |
|---|---|---|---|---|---|---|
| 2015 | June | 217 | 8 | 7 | 0 | 1.4% |
| | September | 133 | 2 | 5 | 0 | 2.3% |
| 2016 | June | 357 | 8 | 15 | 0 | 0.9% |
| | September | 27 | 0 | 0 | 0 | 11.0% |
| 2017 | June | 175 | 11 | 13 | 0 | 1.7% |
| | September | 94 | 3 | 1 | 0 | 3.2% |

**Supplementary Material 2. Meteorological data from 2012 to 2018**

At the meteorological station in Munster (14 km from our site, altitude 420 m, data from Météo-France), the average daily temperatures varied from -13.4°C to 29.3°C between 2012 and 2018 (Figures S2 & S3). The coldest months were in February 2012 (mean: -2.4°C, minimum: -13.4°C maximum: 7.6°C) and January 2017 (mean: -2.7°C, minimum: -9.1°C, maximum: 6.1°C). Early spring (March, April) was particularly hot and dry in 2014 compared to other years and to the average temperatures observed from 1980 to 2010. Early spring 2017 was also hot and dry, with average daily temperatures varying from 8 to 16°C and the 5-day moving average of daily temperature reaching the threshold of 10°C for larvae's questing activity [1,2] at the end of March. However, mid-April was then marked by a rapid drop in temperatures accompanied by snowfall, the minimum temperature reaching -5°C (Figure S2). The summer and autumn were particularly hot in 2015. In July and August 2015, the maximal daily temperatures varied from 18.4 to 38.3°C (median 28.2°C), reaching the highest recorded temperatures since 1986, and the average daily relative humidity varied from 41% to 85% (median 60%) (Figures S3 & S4). The saturation deficit, which integrates temperature and relative humidity to derive a measurement of the drying power of the atmosphere [1,3], was therefore particularly high in summer 2015, varying from 2.3 to 16.2 mmHg (median 7.0, Figure S4). Considering that a saturation deficit < 5 mmHg is favourable for tick behaviour and development, while long-lasting saturation deficit values >10 mmHg may have a negative effect on tick survival [2,4], the saturation deficit was higher than 7 mmHg for 36 days and higher than 10 mmHg for 14 days in the summer of 2015. The autumnal cooling rate estimated by linear regression of the average daily temperatures from 1st August to 31st October was the lowest in 2014 given the mild temperatures in August 2014 (Figure S1).

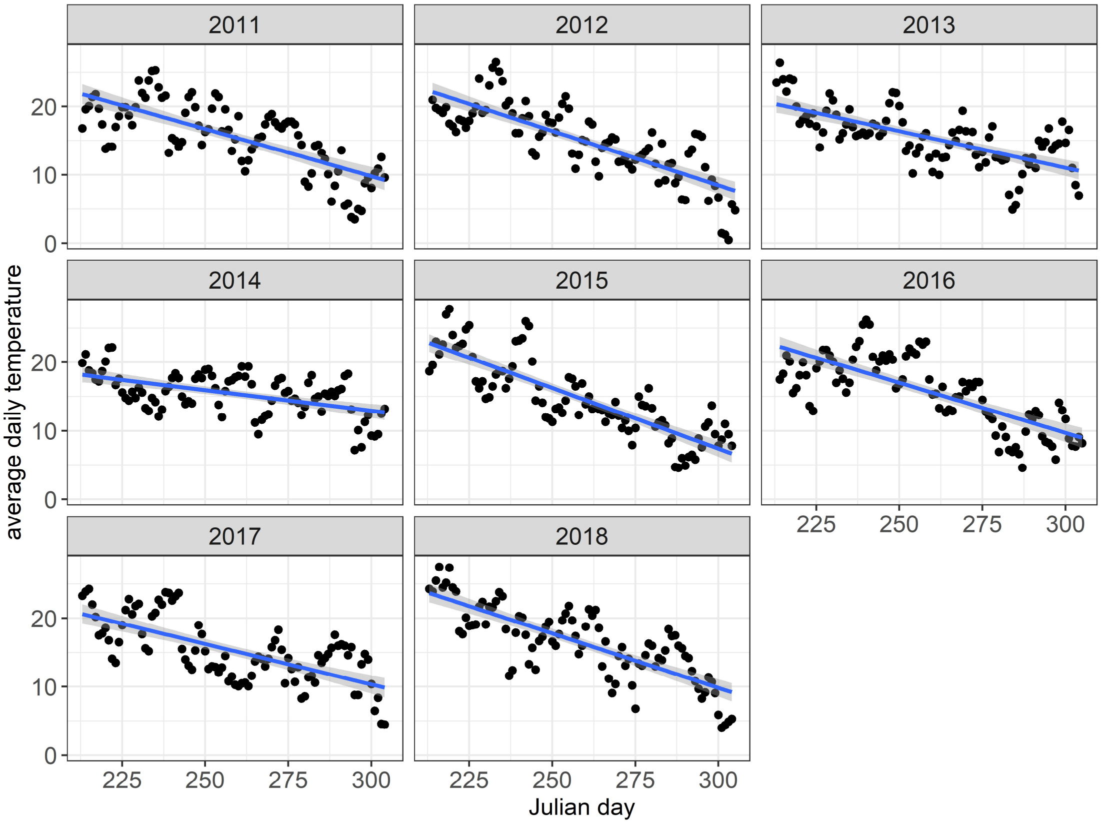


**Figure S1a**: Autumnal cooling rate of temperatures estimated by linear regression of the average daily temperatures from 1st August (Julian day 214) to 31st October (Julian day 305). Source: Météo-France, Munster station.

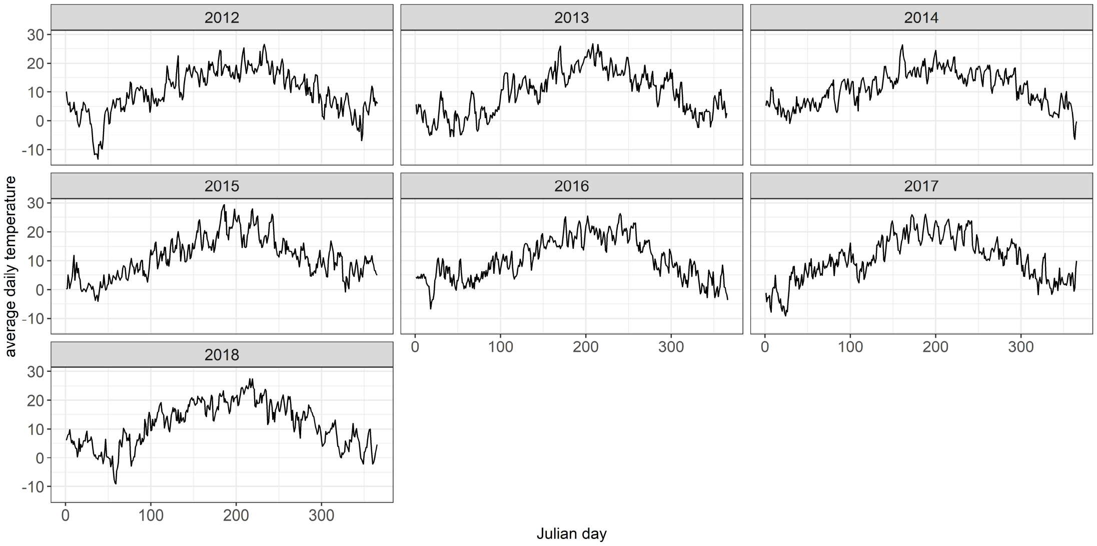


**Figure S1b**: Average daily temperatures per Julian day from 2012 to 2018. Source: Météo-France, Munster station.

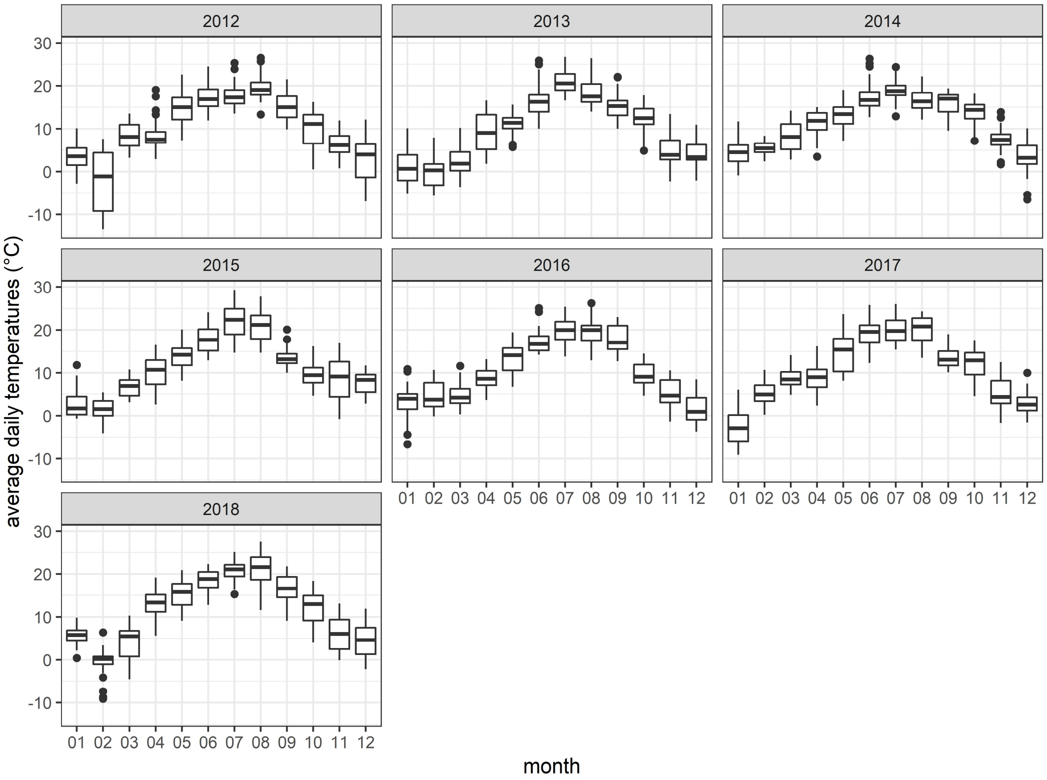


**Figure S1c.** Boxplot of the average daily temperatures per month from 2012 to 2018. Source: Météo-France, Munster station.

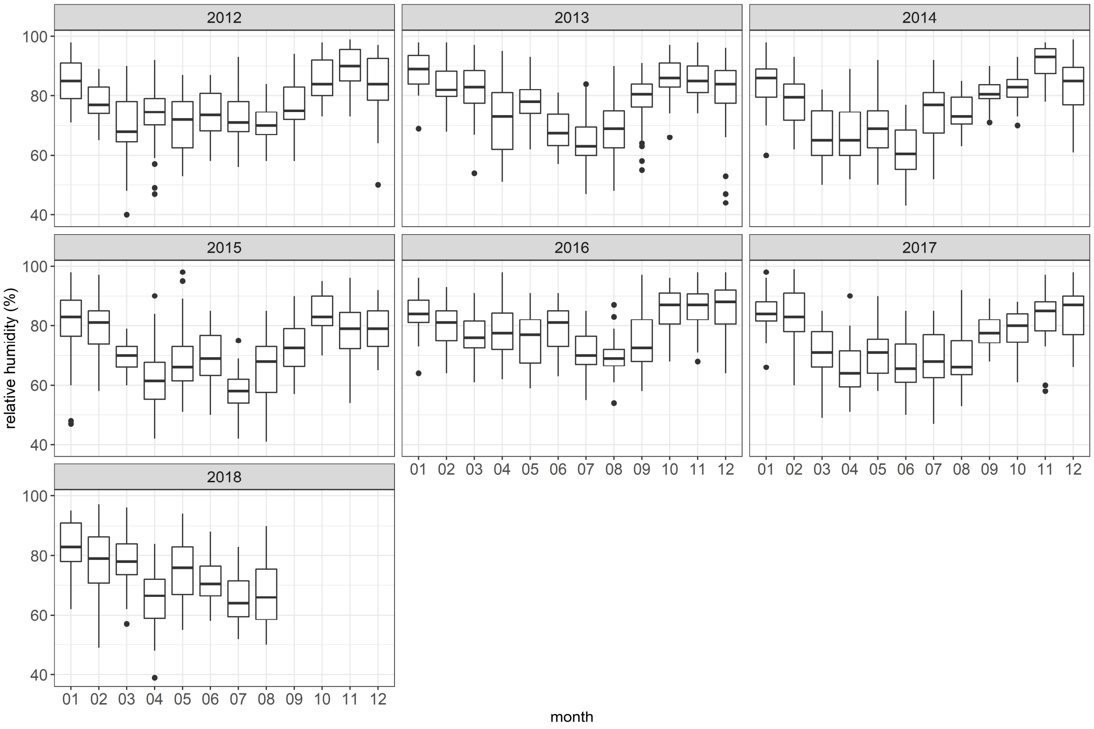


**Figure S1d**: Boxplot of the average relative humidity per month from 2012 to 2018. Source: Météo-France, Munster station. Data were not available for September to December 2018.

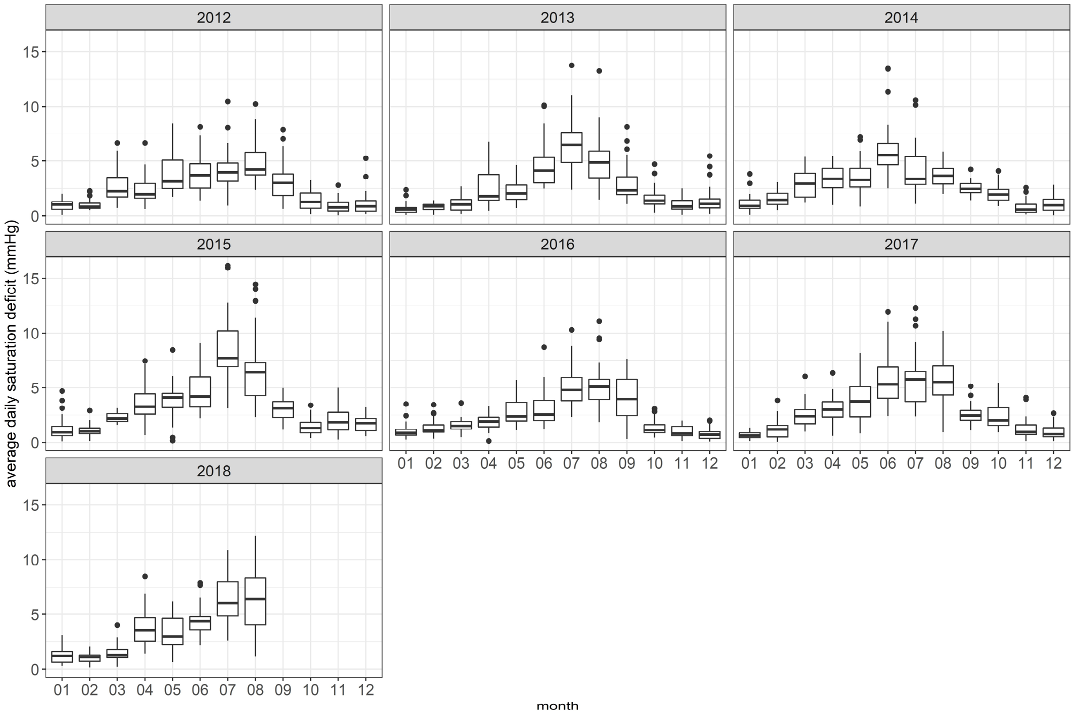


**Figure S1e**: Boxplot of the average saturation deficit per month from 2012 to 2018 (calculated according to the saturation deficit formula of Randolph & Storey, 1999). Source: Météo-France, Munster station. Data were not available for September to December 2018.

## Supplementary Material 3. Relative density indices of deer populations from 2012 to 2018

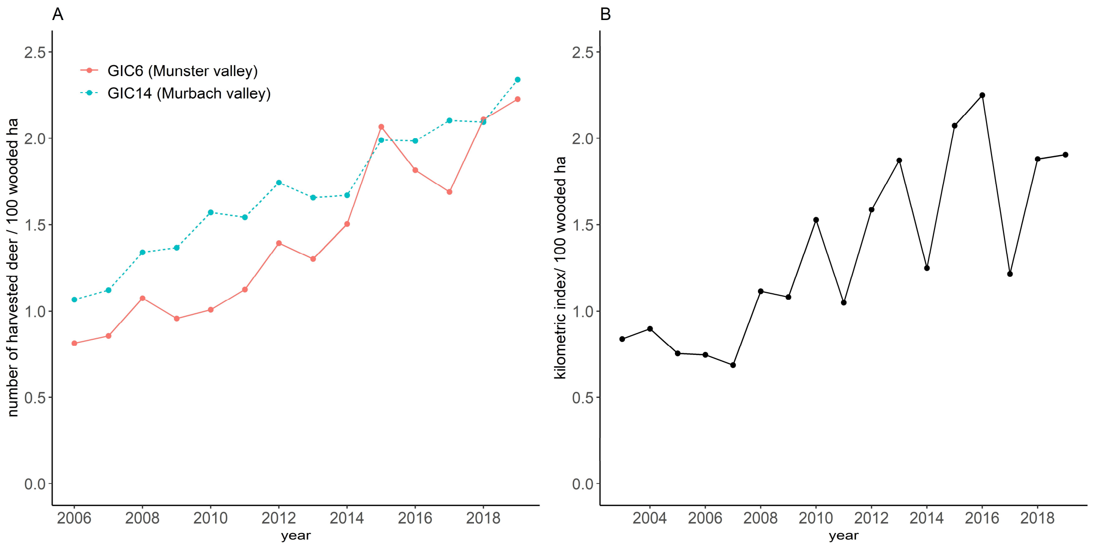


**Figure S2.** Trends of the relative density indices of deer populations in two adjoining hunting units (known as "GICs"), GIC14 including the Murbach study area and GIC6 included the Munster valley. (A) Number of harvested deer per 100 wooded ha in GIC6 and GIC14 from 2006 to 2019. (B) Kilometric Abundance Index in GIC6 per 100 wooded ha (mean number of deer seen per kilometre when walking across all the transects within the area) from 2004 to 2019. Source: DDT68, FDC68.

## Supplementary Material 4. Distribution of human TBE cases in Alsace

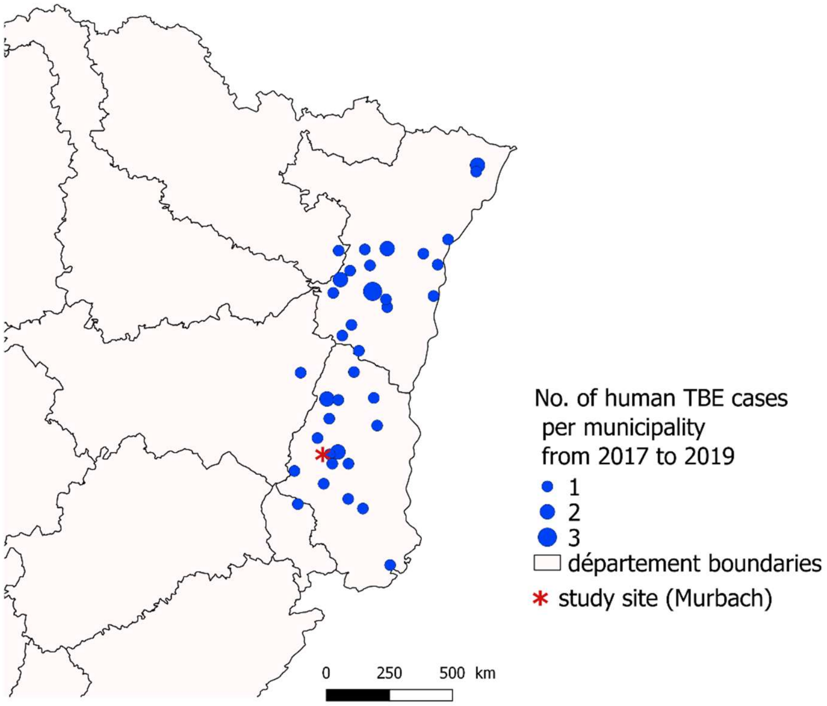


**Figure S3.** Distribution of human tick-borne encephalitis cases in Alsace from 2017 to 2019. Source: Velay & Hansmann, CHU Strasbourg.

## References


1. Perret, J.-L.; Guigoz, E.; Rais, O.; Gern, L. Influence of saturation deficit and temperature on *Ixodes ricinus* tick questing activity in a Lyme borreliosis-endemic area (Switzerland). *Parasitol. Res.* **2000**, *86*, 554–557, doi:10.1007/s004360000209.
2. Tagliapietra, V.; Rosà, R.; Arnoldi, D.; Cagnacci, F.; Capelli, G.; Montarsi, F.; Hauffe, H.C.; Rizzoli, A. Saturation deficit and deer density affect questing activity and local abundance of *Ixodes ricinus* (Acari, Ixodidae) in Italy. *Vet. Parasitol.* **2011**, *183*, 114–124, doi:10.1016/j.vetpar.2011.07.022.
3. Randolph, S.E.; Storey, K. Impact of microclimate on immature tick-rodent host interactions (Acari: Ixodidae): implications for parasite transmission. *J. Med. Entomol.* **1999**, *36*, 741–748, doi:10.1093/jmedent/36.6.741.
4. Gern, L.; Morán Cadenas, F.; Burri, C. Influence of some climatic factors on *Ixodes ricinus* ticks studied along altitudinal gradients in two geographic regions in Switzerland. *Int. J. Med. Microbiol.* **2008**, *298*, 55–59, doi:10.1016/j.ijmm.2008.01.005.
5. Bournez, L.; Umhang, G.; Moinet, M.; Richomme, C.; Demerson, J.-M.; Caillot, C.; Devillers, E.; Boucher, J.-M.; Hansmann, Y.; Boué, F.; et al. Tick-borne rncephalitis virus: seasonal and annual variation of epidemiological parameters related to nymph-to-larva transmission and exposure of small mammals. *Pathogens* **2020**, *9*, 518, doi:10.3390/pathogens9070518.
6. Schwaiger, M.; Cassinotti, P. Development of a quantitative real-time RT-PCR assay with internal control for the laboratory detection of tick borne encephalitis virus (TBEV) RNA. *J. Clin. Virol.* **2003**, *27*, 136–145, doi:10.1016/S1386-6532(02)00168-3.
7. Bestehorn, M.; Weigold, S.; Kern, W.V.; Chitimia-Dobler, L.; Mackenstedt, U.; Dobler, G.; Borde, J.P. Phylogenetics of tick-borne encephalitis virus in endemic foci in the upper Rhine region in France and Germany. *PLOS ONE* **2018**, *13*, e0204790, doi:10.1371/journal.pone.0204790.
8. Perez-Eid, C.; Hannoun, C.; Rodhain, F. The Alsatian tick-borne encephalitis focus: presence of the virus among ticks and small mammals. *Eur. J. Epidemiol.* **1992**, *8*, doi:10.1007/BF00144797.
9. Cannon, R.M. Sense and sensitivity — designing surveys based on an imperfect test. *Prev. Vet. Med.* **2001**, *49*, 141–163, doi:10.1016/S0167-5877(01)00184-2.
